# Comparative Evaluation of Debris Extrusion, Remaining Dentin Thickness and Fracture Resistance of Endodontically Treated Teeth Using Rotary and Reciprocating Endodontic File Systems: An In Vitro Study

**DOI:** 10.7759/cureus.42290

**Published:** 2023-07-22

**Authors:** Prakriti Jaggi, Sanjyot Mulay, Anita Tandale, Renuka Jadhao, Poonam Joshi, Sanket Aras, Karishma Krishnakumar, Vamshi Krishna

**Affiliations:** 1 Department of Conservative Dentistry and Endodontics, Dr. D.Y. Patil Dental College and Hospital, Dr. D.Y. Patil Vidyapeeth, Pune, IND

**Keywords:** fracture resistance, remaining dentin thickness, debris extrusion, waveone gold, trunatomy, root canal preparation

## Abstract

Background: Preventing the apical extrusion of debris during instrumentation is of paramount importance to reduce the occurrence of flare-ups in endodontically treated teeth. Furthermore, an essential requirement for the longevity and strength of an endodontically treated tooth and its ability to resist fracture is the preservation of residual dentin thickness during instrumentation. The aim of this study was to compare the amount of debris extrusion, remaining dentin thickness at the coronal third, middle third, apical third, and the fracture resistance of the teeth using rotary (TruNatomy; Dentsply Sirona, Charlotte, NC) and reciprocating (WaveOne Gold; Dentsply Sirona) endodontic file systems.

Methods: An in vitro study included 52 single-rooted, oval canal shaped teeth that underwent exploration and initial cleaning with a no. 15 K-file. The prepared canals were then randomly assigned to two groups: Group I, with instrumentation with the TruNatomy rotary file (n=26) and Group II, with instrumentation with the WaveOne Gold reciprocating file. Parameters like debris extrusion, remaining dentin thickness, and fracture resistance were evaluated in both the groups. Analysis was performed using a paired t*-*test for the assessment of difference between groups and one-way ANOVA test followed by the post-hoc Tukey test for difference between the coronal, middle and apical third for each study group.

Results: The results revealed no significant difference (t=0.454, p=0.652) between the TruNatomy rotary file and WaveOne Gold reciprocating file in apical extrusion of debris after their use in root canal therapy. For the remaining dentin thickness, a significant difference was present between the TruNatomy rotary file and WaveOne Gold reciprocating rotary file at the coronal (t=5.766, p<0.0001) and middle (t=3.690, p=0.001) levels. The mean fracture resistance was significantly more (t=15.877, p<0.0001) with the TruNatomy rotary file compared to the WaveOne Gold reciprocating rotary file.

Conclusion: The TruNatomy rotary file system outperformed the WaveOne Gold reciprocating file system in terms of maintaining the remaining dentin thickness and providing improved fracture resistance. Nevertheless, debris extruded apically was comparable between the TruNatomy rotary file system and the WaveOne Gold reciprocating file system.

## Introduction

Successful root canal therapy is based on the therapeutic triad of comprehensive canal debridement, efficient disinfection, and complete obturation with the apical and coronal seal [1]. The chemomechanical preparation of the root canal enables one to achieve these endodontic therapeutic goals [2].^ ^The literature reports on working length estimation and root canal preparation 0.5-1 mm short of the apex [3]. However, there is still a controversy with regard to the termination point in preparation length as few authors have also suggested a complete canal preparation till the apex to avoid re-infection following root canal therapy, while few suggested a greater possibility of debris extrusion if the apical limit is pushed beyond 1 mm short to the apex [4].

During the canal preparation process, the irrigants, dental debris, bacteria, necrotic pulp tissue, and their by-products might protrude beyond the apex, and may create additional challenges such as pain, flare-ups, and a delay in periapical tissue healing with an incidence of 1.4%-1.6% according to studies [5-7]. Moreover, the extrusion is significantly more in cases of instrumentation beyond the termination point [3]. Among the various factors responsible for extrusion such as canal size, type of tooth, the curvature of the canal, the technique of preparation, irrigation solution used, and the delivery system, the type and design of the instrument used, its kinematics, and the number of files used play a vital role [8]. The debate continues on the superiority of rotary instruments over hand files to limit the debris extrusion. However, continued research and innovation in the instrument design and technique has led to the development of single-file systems and reciprocating files that have not just simplified the process but also declined the use of multi-step chemomechanical preparation [9]. The evidence highlights the findings of less debris generation with rotational motion over push-pull instrumentation as the rotary file tends to pull the dentinal debris into its flutes that is then directed towards the coronal end. Similarly, the crown-down technique compared to the linear filing motion technique has been found to create less apical extrusion as the crown-down technique aids in expelling the debris from the coronal orifice [3].

The remaining dentin thickness in an endodontically treated tooth is one of the most critical parameters for its longevity because it impacts the resistance of the tooth to fracture. A significant clinical failure during or following root canal treatment is vertical root fracture, with its reported prevalence across the studies ranging from 16.42% to 18.7% [10-12]. The radius of the canal curvature, size and form, as well as the external morphology, all are contributing factors towards the risk of fracture. A reduced dentin thickness tends to increase the magnitude of tensile stress [13]. Finite element analysis demonstrated canal curvature to be more significantly associated with fracture resistance of teeth than external root morphology [14].

To overcome the concern of excessive dentin removal while instrumentation, a number of nickel-titanium (NiTi) instrument systems were introduced with the intention of improving the preparation of curved canals, minimising anatomical deformities and iatrogenic error hazards [15]. Furthermore, the concept of taper of endodontic instruments has shifted from 6% to 4%, with a focus on minimally invasive approaches. The 4% taper rotary instruments exhibit less dentin removal than the 6% taper, resulting in improved fracture resistance [16]. The TruNatomy NiTi file system is employed in continuous rotation while the WaveOne Gold NiTi file system allows for reciprocating back and forth movement. Both these file systems have advantage of increasing the fracture resistance by maintaining an adequate dentin thickness. The present study aimed to evaluate and compare the amount of debris extrusion, remaining dentin thickness at the coronal third, middle third, apical third, and the fracture resistance of teeth using rotary (TruNatomy) and reciprocating (WaveOne Gold) endodontic file systems.

## Materials and methods

This in vitro study was conducted at Dr. D. Y. Patil Dental College and Hospital, Dr. D. Y. Patil Vidyapeeth, Pune. The study included 52 extracted single-rooted mandibular premolars with oval canals. The sample size was calculated using G*Power 3.1.9.2 software, with alpha error 0.05, power 80% and effective size 0.80. After extraction, teeth were cleaned and stored in a 0.1% thymol solution until use. The presence of oval canals was confirmed by capturing radiographs of the teeth in buccolingual and mesiodistal directions.

Tooth preparation

This was performed by a single operator. The access cavity was prepared with a small round diamond bur, BR-45 (Mani); the working length was determined by inserting a no. 15 K-file to the root canal terminus and subtracting 1 mm from this measurement. The tooth was then positioned in a custom-made specimen holder and scanned using a cone beam CT (CBCT) scanner. The prepared canals were then randomly assigned to two groups according to the convenience sampling method: Group I (n=26), with instrumentation with the TruNatomy rotary file (Dentsply Sirona, Charlotte, NC), and Group II (n=26), with instrumentation with the WaveOne Gold reciprocating file (Dentsply Sirona), for the assessment of debris extrusion, remaining dentin thickness, and fracture resistance.

Assessment of debris extrusion

Eppendorf tubes were inserted into the glass vials and the roots were fixed in rubber plugs of the vials with cyanoacrylate. A 21-gauge needle was inserted into the rubber plugs to equalize the outside and inside pressure.

Instrumentation of canals was done as per manufacturer’s instruction: for TruNatomy, 500 rpm with torque of 1.5 Newton centimeters; for WaveOne Gold, 350 rpm speed in 150° counterclockwise (CCW) and 30° CW, turning 360° after three cycles of reciprocation. The tip size and taper of TruNatomy was 26 and 0.04, respectively, while the tip size and taper of WaveOne Gold was 35 and 0.06, respectively. Intermittent irrigation was done with normal saline followed by copious final irrigation with 17% ethylenediaminetetraacetic acid (EDTA).

The specimens were then detached from the apparatus and placed in a hot air oven at 140°C for five hours to evaporate the solution [17]. The pre-weight of Eppendorf tubes was measured three times before being placed in the vials and the final weight was obtained by weighing three times and taking the mean values. The weight of the extruded debris was calculated by subtracting the pre-weight from post-weight.

Assessment of the remaining dentin thickness

Post-instrumentation CBCT scans were taken and the thickness was calculated at 3 mm (apical third), 6 mm (middle third), and 9 mm (coronal third) from the apex. The formula (X1 - X2) - (Y1 - Y2) was used for calculating the remaining dentin thickness, where X1 was the shortest distance from the mesial edge of the root to the mesial edge of the un-instrumented canal, Y1 was the shortest distance from the distal edge of the root to the distal edge of the un-instrumented canal, X2 was the shortest distance from the mesial edge of the root to the mesial edge of the instrumented canal, and Y2 was the shortest distance from the distal edge of the root to the distal edge of the instrumented canal [18].

Assessment of fracture resistance

Canals were obturated with the warm vertical compaction technique using gutta percha and AH Plus sealer (Dentsply Sirona) followed by post-endodontic restoration with composite resin. The teeth were then fractured using a universal testing machine running at a crosshead speed of 1 mm/min. The load at the fracture was recorded in kilograms force and the type of fracture was noted.

**Figure 1:**
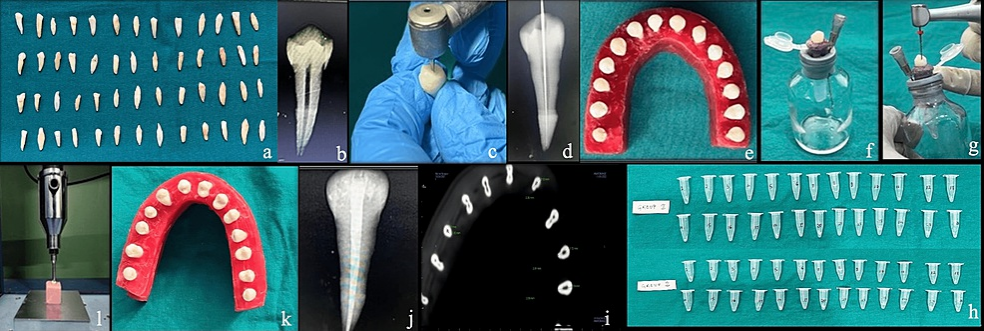
Pictorial representation of the study procedure (a) Extracted single-rooted teeth. (b) A digital radiograph was taken to confirm the single oval canal. (c) Access opening was done. (d) Working length was confirmed radiographically. (e) Teeth were mounted on wax and CBCT was performed. (f) Each tooth was fixed in the rubber plug of the glass vial with cyanoacrylate. (g) Root canal instrumentation was done. (h) Eppendorf tubes showing apically extruded debris. (i) CBCT was done after instrumentation and the remaining dentin thickness was calculated using Invivo software (Dexis, Quakertown, PA). (j) Canals were dried, and obturation was done. (k) Post-endodontic restoration was done with composite resin. (l) Each tooth was embedded in a putty impression material and a continuous load was delivered through a universal testing machine until fracture occurred. CBCT, cone beam CT

The study procedure is shown in Figure 1.

Statistical analysis

The data obtained was analysed using SPSS Statistics, version 21 (IBM Corp., Armonk, NY). The normality of the data was assessed using the Kolmogorov-Smirnov test. The difference in debris extrusion, remaining dentin thickness, and fracture resistance between the groups was analysed using a paired t-test while the difference between the coronal, middle and apical third for each group was analysed using the one-Way ANOVA test followed by a post-hoc Tukey test. The significance was kept at p<0.05.

## Results

The results revealed no significant difference (t=0.454, p=0.652) between the TruNatomy rotary file and the WaveOne Gold reciprocating file in apical extrusion of debris after their use in root canal therapy (Table 1).

**Table 1:** Difference in the mean debris extrusion after the use of TruNatomy and WaveOne Gold reciprocating rotary files

Groups	N	Mean ± standard deviation	Mean difference	t-value	Significance (p)
TruNatomy group	26	0.8350 ± 0.01	0.00168	0.454	0.652
WaveOne Gold reciprocating group	26	0.8367 ± 0.02			

Regarding the intragroup comparison of the remaining dentin thickness, there was no significant difference between the coronal, middle, and apical level when cleaning and shaping was performed with the TruNatomy rotary file (F=2.755, p=0.073) and WaveOne Gold reciprocating rotary file (F=0.533, p=0.590). Nevertheless, a significant difference was present in the intergroup comparison of the TruNatomy rotary file and WaveOne Gold reciprocating rotary file at the coronal (t=5.766, p<0.0001) and middle level (t=3.690, p=0.001) of the root indicating the remaining dentin thickness to be significantly more with the TruNatomy rotary file compared to the WaveOne Gold reciprocating rotary file. However, there was no statistical difference in the apical third of the study groups (t=0.528, p=0.600) (Table 2).

**Table 2:** Mean difference in the remaining dentin thickness at the coronal, middle and apical third between the TruNatomy rotary group and WaveOne Gold reciprocating rotary file group *Significant

Level	Groups	Mean ± standard deviation	Mean difference	t-value	Significance (p)
Coronal	TruNatomy	0.1904 ± 0.05	0.09692	5.766	<0.0001*
	WaveOne Gold reciprocating	0.0935 ± 0.07			
Middle	TruNatomy	0.1719 ± 0.05	0.07500	3.690	0.001*
	WaveOne Gold reciprocating	0.0969 ± 0.09			
Apical	TruNatomy	0.1288 ± 0.10	0.01615	0.528	0.600
	WaveOne Gold reciprocating	0.1127 ± 0.12			

The mean fracture resistance after the endodontic procedure was significantly more (t=15.877, p<0.0001) with the TruNatomy rotary file compared to the WaveOne Gold reciprocating rotary file. For all three parameters, the TruNatomy rotary file significantly outperformed the WaveOne Gold reciprocating rotary file (Table 3).

**Table 3:** Difference in the mean fracture resistance in TruNatomy and WaveOne Gold reciprocating rotary file groups *Significant

Groups	N	Mean ± standard deviation	Mean difference	t-value	Significance (p)
TruNatomy	26	298.60 ± 18.52	64.94	15.877	<0.0001*
WaveOne	26	233.66 ± 9.60			

## Discussion

The success of root canal therapy depends on the thorough removal of pulpal tissue, dentinal debris, and biofilm, as well as adequate bio-chemo-mechanical tooth preparation. Additionally, a sterile environment inside the canal with a complete 3D hermetic seal with the biocompatible obturating substance is equally essential [19]. The advancements in rotary NiTi files have led to a great improvement in the cleaning and shaping procedures. In the present study, the WaveOne Gold reciprocating file system and the TruNatomy rotary file system were used in cleaning and shaping to determine the superiority of one file system over the other for debris extrusion, remaining dentin thickness, and fracture resistance of teeth. There was no significant difference between the TruNatomy file group and the WaveOne Gold file group in terms of mean debris extrusion after their use. However, the TruNatomy file system demonstrated less debris extrusion in comparison to the WaveOne Gold file. In a study by Waleed et al., TruNatomy outperformed all other experimental groups in terms of cleaning ability, and exhibited improved cleanliness at the coronal and middle third of the roots [20].

The results of the present study displayed a contrast with the study by Roshdy and Hassan wherein the WaveOne Gold file system outperformed the TruNatomy system in severely curved canals [21]. Similarly, Genius (Ultradent Products, Inc., South Jordan, UT) and EdgeEndo X7 system (EdgeEndo, LLC, Albuquerque, NM) with a reciprocating mechanism were associated with less apically extruded debris when compared to the continuous rotation file system [22]. These studies highlighted the balanced force and pressure-less mechanics to be the reason of the excellent outcome with the reciprocating file system. Moreover, varying results across the literature can be attributed to the difference in the methodologies, tooth samples used, irrigating solutions, and the kinematics of the instruments. The primary reason for flare-ups, postoperative discomfort, and complications following endodontic therapy is reported to be apical debris extrusion during chemomechanical preparation of teeth [23],^ ^wherein instrumentation methods, master apical instrument size, endodontic file speed and torque, and instrument design contribute towards the mechanical component while the apical constriction architecture, root dentin hardness, fluid flow within the root canal, and the position of the tooth contribute towards the natural physical elements [24].

In the present study, no significant difference within groups was observed at the coronal, middle and apical third with respect to the remaining dentin thickness after instrumentation within the TruNatomy group and WaveOne Gold group. However, the remaining dentin thickness was significantly more in the TruNatomy rotary file group compared to the WaveOne Gold reciprocating rotary file group. Three levels, i.e., cervical, middle and apical third of root canals, were chosen in the present study for evaluation as they represent the area with chances of high vulnerability leading to iatrogenic mishaps [25]. The results were in accordance with the study by Marceliano-Alves et al. that indicated no differences in the apical third region across the groups and that Reciproc (VDW Dental, Munich) and WaveOne were linked to noticeably larger differences than the other groups with Twisted File (Kerr, Brea, CA) and HyFlex CM (Coltene Whaledent, Altstätten, Switzerland) systems [26]. A case report further supported the results of the present study and stated that TruNatomy preserves the natural canal integrity, dentine thickness, and maintains the structural integrity of teeth. The TruNatomy system provides the clinicians with superior debridement by respecting the original canal structure, with an emphasis on advantages such as dentine preservation, increased performance, and effectiveness [27]. Additionally, the TruNatomy file system also demonstrated promising results in double-curved simulated canals and removed less resin. The study emphasized on the role of apical taper in the causing of canal transportation in the apical curvature area [28].

It should be noted that dentinal abnormalities like craze, cracks, and ultimately, vertical root fractures of teeth are associated with all Ni-Ti files, regardless of motion kinematics [19]. However, the incidence of dentinal defects is lower with instruments functioning in continuous rotation than with instruments functioning in reciprocating motion [16].^ ^Furthermore, the superiority of TruNatomy over other file systems can be attributed to the lesser taper design, dentin-preserving capability and canal-centering ability of the file.

The objective of measuring fracture strength, which involves continuously placing external stress on the root until it breaks, is to ascertain how weak the root is as a result of endodontic procedures. The instrumentation procedures during the root canal therapy form the craze lines and cracks that result in vertical root fractures [29]. Furthermore, a tooth is more vulnerable to fracture when the canal diameter exceeds 40% of the root width, and the tooth becomes weaker and more prone to fracture under functional loads when access is made through the pulp chamber, along with the chamber walls and canal orifices. Thus, fracture resistance is directly related to the remaining dentin thickness of teeth after biomechanical preparation [26-30], and secondarily to the instrumentation of canals, dentin dehydration, and the uncontrolled pressure applied during obturation [31].

The mean fracture resistance in the present study was significantly more in TruNatomy rotary file group compared to the WaveOne Gold reciprocating file group. The results were in agreement with those of Morales et al. that stated that TruNatomy maintained canal anatomy better than WaveOne Gold by touching the lowest percentage of the canal surface. This resulted in a greater amount of remaining dentin thickness and ultimately higher fracture resistance [32]. Similarly, the canals instrumented with TruNatomy files showed the most significant resistance towards fracture when compared to WaveOne in single-rooted premolar teeth. The superior nature of TruNatomy can be attributed to the off-center parallelogram cross section of the file that conserved more dentin thickness thereby increasing the durability and resiliency of teeth that have undergone endodontic treatment [33]. In the study by Falakaloglu et al., TruNatomy was also shown to have less apical extrusion of debris compared to ProTaper Gold (Dentsply Sirona) in single-rooted teeth. Furthermore, TruNatomy is available with the taper of 0.08, 0.02, 0.04, 0.03, and 0.04 with an orifice modifier file, a glider file, prime file, medium file, and a small file, respectively, that can be used as per the canal morphology for efficient debris removal and optimal shaping of the root canal while preserving the structural integrity [34]. Likewise, WaveOne Gold is described to have a progressive taper of 0.07, 0.06, and 0.05 for primary, medium, and large files that help to efficiently shape the root canal. Adding to this, the WaveOne Gold files have an alternating offset parallelogram-shaped cross section that efficiently remove dentin and shape the canal walls [35].

Our study also has certain limitations. First, the sample size was small. Second, the study comes with the limitations of an in vitro study design recommending the clinical investigations in an in vivo simulated environment.

## Conclusions

Within the limitations of the study, it is concluded that the TruNatomy rotary file system outperformed the WaveOne Gold reciprocating file system in terms of maintaining the remaining dentin thickness and providing improved fracture resistance. However, the amount of debris extruded apically was comparable between the TruNatomy rotary file system and the WaveOne Gold reciprocating file system. It can also be concluded that the TruNatomy rotary file system was the best in preserving the pericervical dentin, and hence, fracture resistance was increased compared to the WaveOne Gold reciprocating file system. However, further in vivo studies are required to support the results.
